# Prophylaxis of Social Diseases1Read by invitation before the State Medical Society of Maryland, the State Conference of Charities and State Federation of Women’s Clubs.

**Published:** 1907-10

**Authors:** Prince A. Morrow


### Prophylaxis of Social Diseases.[1]

By PRINCE A. MORROW, A. M., M. D.

(*Author's Abstract—Maryland Medical Journal, September, 1907.*)

I WISH to express my appreciation of the honor of being invited to appear before this representative body of the medical profession, members of the State Conference of Charities, and of the State Federation of Women's Clubs. It is a source of especial gratification to find so many men and women engaged in different spheres of social activity, uniting with medical men in the discussion of a problem of preventive medicine which has such important relations with the interests of the social welfare. This composite gathering exemplifies the solidarity—the community of interest and responsibility existing between the medical profession and social workers in all questions relating to the physical and moral health of the community. The office of hygiene is not limited to the care of the health of individuals; its broader function is to develop all those conditions which conduce to public health and which in its highest expression is inseparable from public morality.

It is generally conceded that medicine constitutes the most important department of human knowledge, and its value to humanity is largely measured by the degree in which it is applied to the prevention of disease.

Those of you whose charitable activities are directed to the relief of the dependent members of society, cannot fail to recognise that disease is one of the most important factors in the causation of the destitution which requires relief. The prevention of disease, which transforms the bread winner into the dependent upon charity, has a most important economic as well as humanitarian value.

The medical profession has long recognised that the fight against communicable diseases is not simply a struggle against microbes, but a warfare, as well, against bad social conditions, and, further, that the conquest of these diseases is not possible

---

1. Read by invitation before the State Medical Society of Maryland, the State Conference of Charities and State Federation of Women's Clubs.

without the aid and co-operation of social agencies which can effectively intervene in the correction of the bad social conditions through which disease germs are spread. The value of the combination of medical and social agencies has been most signally shown in the warfare which is now being waged against the Great White Plague; its success has been rendered possible only by the education of the public and the effective aid of social workers and public-spirited citisens in the improvement of the housing and living conditions of the people.

With tuberculosis, perhaps more than tuberculosis, social diseases constitute the greatest social scourge of our modern civilisation. This class of diseases has been aptly designated "The Great Black Plague." Working in darkness and disguise, protected by their privacy, their shame and their secrecy, they infect, unseen, the social body. Without the pale of public interest or sympathy, unfettered by any semblance of sanitary control, they have been practically abandoned to their own evolution. Their neglect has always been considered the reproach and their prevention the despair of sanitary science.

It is a sign of progress and a hopeful augury of success that men and women representing influential social organisations have signified by their presence here today their willingness to join forces with the medical profession in a socio-sanitary movement which, it is hoped, will limit at least the diseases we wish to prevent.

It is eminently fitting that women should interest themselves in this movement for the prophylaxis of social diseases. It is upon women that the burden of shame and suffering, of disease and death, is chiefly laid—not so much, perhaps, upon that unfortunate class who are regarded as the chief agents in the propagation of these diseases, but upon pure women, who do not always find, even in the sanctuary of marriage, a safeguard against "the diseases of the women of the streets." By a strange irony of fate, the diseases of vice transplanted to the bed of virtue often become intensified in virulence and danger; their worst effects are developed in fulfilling the functions for which marriage is instituted. It is not alone upon the virtuous wife, but upon the children who are a part of her being that the blighting, destructive force of this social scourge most heavily falls.

Before an exclusively medical audience it would scarcely be necessary to refer to the pathological significance of the class of infections comprehended under the general term "social diseases," but before a mixed audience brief reference may be made to their extensive prevalence and their dangers to the individual and society in order to emphasise the importance of the prophylactic work which it is hoped may be inaugurated in this city.

As these diseases are not subject to official registration, there are no available statistics which enable us to formulate the amount of venereal morbidity in this country. Competent European observers state that 75 per cent. of the adult male population have or have had gonorrhea, and 10 to 18 per cent. contract syphilis. It would be a conservative estimate to state that the morbidity from both these infections would represent 60 per cent. of the adult male population in this country. While these diseases may occur at any period of life, they are essentially maladies of early life, probably 60 per cent. of infections occurring before the twenty-fifth year.

The danger of these diseases is measured not only by their effects upon the health or life of the individual, but upon the family and the race. Our conception of their pathological import has been singularly amplified by the acquisitions made to our knowledge within the last third of a century, especially of the serious nature of gonococcus infection in women. Gonorrhea, in addition to its local inflammatory complications, is often the cause of permanent sterility in the male. It has a much wider range of morbid action than was formerly supposed; the gonococci are susceptible of being taken up in the circulation, producing serious and deforming inflammation of the joints and lesions of internal organs which may terminate fatally.

The significance of syphilis as a danger to health and life is not measured so much by its immediate effects as by the changes it sets up in certain internal organs essential to life; such as the brain, liver, heart and arterial system, and which are the cause of death at a more or less remote period. Many of these serious manifestations occur after the tenth year of the disease, and are especially liable to involve the nervous system. It is estimated that 90 per cent. of cases of locomotor ataxia, a large but indeterminate proportion of the paralyses and general paresis are caused by syphilis.

Recent investigations in the French insane hospitals show that 25 to 39 per cent. of deaths in those institutions may be traced to syphilis. The chief significance of these diseases as a social danger comes from their introduction into married life. It is the popular impression that they are spread exclusively through illegitimate sexual relations. Unfortunately, a large proportion of men contract these diseases at or before the marriageable age. Many of them marry, ignorant of the fact that they are bearers of contagion to their wives and offspring. Gynecologists tell us that 80 per cent. of the inflammatory diseases peculiar to women and 50 per cent. of all the operations performed by surgeons on the maternal organs are the result of gonococcus infection. One specific effect of this disease upon the pelvic organs of women is to extinguish the conceptional capacity. It is estimated that 50 per cent. of gonorrheally infected women are rendered permanently sterile.

While gonorrhea is not susceptible of hereditary transmission, it is liable to infect the eyes of the child at birth. Eighty per cent. of the ophthalmia of the new born, and 15 to 20 per cent. of all blindness is attributed to gonococcus infection, to say nothing of the vulvo-vaginitis, the arthritis and other accidental infections of children in family life. If the wife is infected with syphilis, in addition to the risks to her individual health already referred to, the disease may be transmitted in full virulence to the offspring, killing them outright or resulting in physical and mental weaklings. From 60 to 80 per cent. of syphilitic children die before being born or shortly after birth; those that finally survive are subject to various organic defects and degenerative changes, which are susceptible of being transmitted to the third generation. It will thus be seen that diseases of this class, from their specific effect upon the reproductive organs, their damage to the procreative capacity, their deteriorating influence upon the offspring, constitute the most powerful foes to the productivity, the vitality and physical progress of the race.

It was the recognition of the significance of these diseases as a social peril that was the impelling motive to the inauguration of the work undertaken by the American Society of Sanitary and Moral Prophylaxis. It was believed by those who started this movement that it was time to break with the existing policy of silence and inaction, and to organise a social defence against a class of diseases which are most injurious to the highest interests of society. The basis of any intelligent scheme of prophylaxis is the adaptation of its measures to the causes of the disease and the conditions under which it is spread. While sanitary science has been reproached for its utter inefficiency in evolving any practical scheme of control, it is evident that the prevention of social diseases cannot be treated as a purely sanitary problem. Their causes reside in conditions which lie entirely without the sphere of sanitary control, and their communicative mode cannot be reached by repressive measures. It was determined to enlist the co-operation, as far as possible, of all the social forces which could render effective aid in the corrections of the conditions of which these diseases are the outgrowth.

From whatever standpoint this field was surveyed, the indifference of the public, the reckless and voluntary exposures to infection, the marital contaminations, the hereditary horrors, the most obvious causes seemed to converge and center in the focal point of ignorance. The public is indifferent because ignorant of the extent and significance of venereal morbidity; the young who voluntarily expose themselves do not know the veritable danger of these infections, nor the imminence of this danger; the men who carry disease and death into their families are ignorant of the laws of venereal contagion, its varied and multiple modes; they do not know its terrible consequences to their wives and children. While there are other and contributory causes, the basic cause is ignorance. The keynote of this movement, then, was sounded as a campaign of education, a crusade against ignorance. This ignorance on the part of the public is not surprising in view of the fact that both social sentiment and professional ethics have always united to cover up and conceal these diseases; all the educational agencies of our social life are organised upon a basis of silence as to their existence even. On the part of the young, ignorant of the dangers which come from the irregular exercise of the sex function is compulsory; sound sex instruction is forbidden as improper; parents, teachers and scholastic instructors are banded together, a complicity of silence.

The public cannot be expected to seek deliverance from a hidden danger, the gravity of which it is utterly incapable of measuring, and the reality of which it scarcely suspects. The first indication is to turn on the purifying light of publicity, to give to the public a knowledge of the facts which so vitally concern its interests, the extent and dangers of the diseases to the individual and to society, and their modes of contagion, direct and indirect. It is necessary to educate the conscience as well as the intellect of the people in order to create a public sentiment in favor of this work which will lead to an intelligent and active co-operation. It is only by opening up the humanitarian aspects of the situation, by exposing the dangers to the innocent members of society, that the conscience of the public can be touched and aroused to the significance of these social crimes and the moral obligation to aid in their prevention. There is needed not only a change in the apathetic attitude of the public, but a change of traditional ideas, of the mental attitude of the public toward the sex problem—a reversal of the policy of our educational system which now forbids instruction in the laws of life and sex.

The ideal of a good education to which most parents cling is one which entirely ignores the existence of sex, that most important fact of life. All thinking men must recognise that the development of the sex function is intimately associated with the physical, mental and moral growth of the individual. Sex is the physical basis of love, of the family sentiment, even of the existence and prosperity of society. The object of education is to fit the individual for complete living, which includes not only selfpreservation, but self-perpetuation. From earliest infancy instruction is given about the functions of the body essential to its maintenance, the care of the stomach and bowels, what to eat and drink and what to avoid, but no word of advice as to the care or warning as to the abuse or irregular exercise of that function which, from a biologic point of view, is the most important function of the human body. One lesson, indeed, the majority of parents give, viz., that the system of generation is a system of shame. This impression is so grounded and fixed in the mind of youth that it is apt to dominate his mental attitude throughout life. This sex instruction of their sons, so inauspiciously begun, is then committed to haphasard sources, to servants, to older and dissolute companions, to quackish literature, to be completed, too often, by harlots. Our educational program proposes to fill this glaring hiatus in home and scholastic instruction, to take account of the sexual organisation of the individual, the origin of facts of life and sex which are now regarded as forbidden subjects.

As I have elsewhere said, the function of the medical profession is to insist upon the value of this education and supply the requisite knowledge, intrusting its practical application to those who command the facilities and are better qualified by experience and a knowledge of specific methods. This education should be begun early, before sensuality is awakened and the curiosity of youth in regard to the mysteries of life and sex takes on a dangerous turn. Upon this foundation should be built, later, instruction in the physiology and hygiene of sex, which should include the true purpose of the sex function, its essential dignity, and, further, that its impulses should be educated, controlled and directed in a proper channel. Later he should be taught the dangers, both physical and moral, which come from the irregular exercise of the sex function. The high purpose of this education is to teach young men how to live according to the laws of a healthy nature by letting them know what those laws are. It aims to promote clean living by cultivating a right attitude of mind toward the passions and appetites; its essential object is to promote continence as the surest prophylactic against venereal infection. This innovation proposes to substitute sound, sanative and wholesome knowledge of the sex function for the erroneous and demoralising instruction the youth now receives from ignorant and often vicious sources.

Sound knowledge never does harm; it is knowing things wrong that does the mischief. A celebrated Grecian philosopher has said "the most needful piece of learning for the uses of life is to unlearn all that is untrue." This applies with especial force to the existing knowledge of young men of the present generation in sex matters. It is important that the young man who has had no sex instruction except what he has picked up from ignorant or vicious sources should unlearn the untruth "that the sex function is given solely for sensual pleasure;" he should unlearn that "the exercise of this function is essential to his health and that he has a natural right to indulge his sexual impulse as he pleases;" he should unlearn all those physiologic fallacies upon which the sexual necessity and the conventional standard of morality are based, and especially should he unlearn the ethical heresy that one-half of humanity has imperious duties which the other half may repudiate or disclaim.

While proper sex instruction may trench upon the domain of morals, it is not suggested that the physician should usurp the function of the religious or ethical teacher. It is the province of the physician to teach the hygiene of all the functions of the body; it is his duty to warn against the exercise of any function under conditions which cause disease. The irregular exercise of the sex function, whether it is termed "incontinence" or "immorality," is the direct cause of that vast mass of misery and disease we are now considering. If continence in young men is healthful and compatible with the highest physical and mental vigor, if incontinence is the frequent cause of their physical and moral wreckage, it is the duty of the physician to warn against promiscuous cohabitation. "Physicians," declares Dr. Osler, "should be the apostles of continence." The teaching of continence does not imply, as has been asserted, a Pharisaical assumption of superior virtue; it is an impersonal interpretation of the physiologic laws of man's nature as developed by science and confirmed by experience. In the matter of sex relationship the teachings of hygiene and morality are in complete accord.

In thus emphasising the value of instruction in the physiology and hygiene of sex as a chart for the regulation of sexual conduct, we do not undervalue the teaching which properly comes within the province of the clergy. Hygienic teaching should be reinforced by an appeal to the conscience, so that the duty of clean living may be impressed with the force of a moral obligation. We may now inquire what measure of preventive value we may reasonably expect from this education. No one indulges for a moment the illusion that it will prove an infallible corrective of incontinence. It is believed, however, that many young men when fully instructed as to the peril to the body, the mind and the character which comes from licentious living will choose the safer path of continence until they marry. The chief value of general enlightenment will be the safeguarding of marriage from venereal infection. It is inconceivable that the havoc wrought by these diseases in the home and family will continue when men realise the fearful consequences of marrying with an uncured venereal disease. An enlightened public opinion, which is the strongest force in the evolution of the conscience of the race, will no longer tolerate these social crimes.

I have thus dwelt, and I fear at a wearisome length, upon this, the most important feature of our program, because I believe that the chief hope of success in the work before us lies in the hygienic and moral education of the rising generation. Before dismissing this part of the subject, allusion may be made to that cynicism which, masquerading under the guise of common sense, declares that the impelling motive to licentious relations between men and women cannot be restrained by any considerations of health or morality, of consequences to themselves or others. This despair of educational and moral influences would paralyse all the efforts now being made in every department of social life to correct its abuses, to raise its ideals and to promote its welfare.

The prophylactic value of treatment is so evident to medical men that only the briefest mention of this part of the program will be made. Its chief object is to prevent those already infected from infecting others by promptly sterilising sources of contagion. To accomplish this object the provisions made for the treatment of these diseases should be enlarged, and made available to all, not so much in the interest of the patient himself as in the interest of others he might expose to infection.

As the question of the social evil is to form the subject of another paper on the program, I will touch but briefly upon what generally is regarded as the crux of the entire situation. Certainly there can be no intelligent or comprehensive system of prophylaxis framed which ignores the relation of cause and effect between the social evil and social diseases. It is well, however, to clear away a misconception which exists in the minds of many as to the measure of responsibility of public women for the spread of the diseases of vice. In the ordinary conception, the prostitute, with her cortège of infections, is the exclusive cause of their propagation; but, while the prostitute is the chief source, she is by no means the exclusive agency in its spread; she is but the purveyor of the infection; she returns to one or several consumers the infection she has received from another consumer. It is not the prostitute, but her partner, who carries the poison home and distributes it to his family. It is the husband and father who is the responsible cause of the wreckage of the health and lives of innocent women and children.

Now, the responsibility of the male factor in the spread of these diseases has always been minimised. This constitutes the radical defect of reglementation from a sanitary standpoint. No more inefficient or incomplete sanitary measure could be devised than the examination of public women with the view of eliminating sources of contagion while the male factor in the spread of disease is entirely ignored. If the woman's body is found diseased it is withdrawn from circulation until it can be certified as safe for the consumer, while the latter is permitted to contaminate other women without the shadow of control, even to carry the contagion into his own family. Sex may qualify morality, but it does not qualify the laws of contagious disease. The sanitary feature of this system is condemned by its practical results, without reference to objections on moral grounds. This same unilaterality is manifest in the condemnation of woman as the chief offender against morality. All the so-called "moral crusades" are directed against women alone. In the descent upon disorderly houses the unfortunate women are fined and imprisoned, while the men who are there for the same immoral purpose are allowed to go scot free. I have long ago stated my conviction that the reversal of this one-sided policy—treating men and women precisely alike—would break up these houses.

In tracing the essential cause of prostitution we find that, while socio-economic conditions are contributory causes, we must face the fact that the taproot of this evil is grounded in the polygamous proclivities and practices of man. More than the inherited tendencies to vice in certain women, more than the love of finery and luxury, the laziness, the economic dependence, the force of want that impels many of them along the road to ruin, more than all these and other alleged conditions, the chief cause is the unbridled instinct of man, which, in seeking the means of its gratification, creates the supply to satisfy the demand. The prostitute is largely the creature of man's sensual appetite. The methods of dealing with the social evil have been based upon a recognition of this demand as a necessity for men, and they fail because they endeavor to correct the effects without touching the cause.

As the work of the society has thus far been directed chiefly along educational lines, it can hardly be said to have a definite policy in dealing with the social evil, except that it rejects all measures which ignore the moral issues involved. My own personal view is that this problem should be approached through educational and moral influences rather than by legislative intervention. Efforts should be directed not to making prostitution safe, but to prevent the making of prostitutes. The first indication is to lessen the demand by influences and agencies which act upon the intelligence and moral sense of the individual, by education in the law of sex which teaches that the sexual instinct should be educated, restrained by reason and directed into a monogamous channel, by exposing the danger to the physical health and moral character which are inseparable from licentious relations—dangers which may destroy his reproductive powers or blight the health of his children. This instruction would be incomplete without impressing upon young men that the use of alcohol is one of the most powerful of all influences inciting the sexual debauch.

The second indication is to curtail the sources of supply by throwing additional safeguards around young women of the working classes and the large population of homeless and friendless girls, from which the ranks of prostitution are chiefly recruited. This may be done through education and the aid of those social agencies which have been organised for the protection of young women. A vigorous and unrelenting fight should be made against the purveyors of prostitution—the white-slave trade, the cadet system, the employment agencies, personals in the newspapers—against proxentism in all its forms. Quack advertisements should be suppressed as one of the most powerful agencies in the spread of venereal infection. They minimise its dangers, and, by giving deceptive assurance of cure, the victim goes on spreading the germs of danger in ignorance that he is the bearer of contagion.

In conclusion, brief reference may be made to the experimental work done by the Society of Sanitary and Moral Prophylaxis since its organisation in New York over two years ago. It was recognised that this was a new and untried field, and it was necessary, first of all, to study the situation. Since the evil we wish to correct is largely the result of ignorance, the first indication seemed to be the enlightenment of the public as to the magnitude and significance of the evil. The work of publicity has been especially difficult because of the uncompromisingly adverse attitude of the newspaper press, which excludes all mention of this class of diseases. Through one channel or another, however, this knowledge is generally getting into circulation. The next indication appeared to be the education of the rising generation in sex matters. Sex instruction, which contemplated an innovation upon the established educational system, was recognised to be not only delicate, but exceedingly difficult, as it ran counter to deeply-rooted prejudice, as well as traditional custom. Much study has been given to the character and scope of this education, the age at which it must be given and the agencies through which it should be imparted. The education of the young men and women of the working classes, and the men of the army and navy service, has also been the subject of careful study. The subject of throwing additional safeguards around marriage, ethical as well as legal, has been considered. The results of these studies appear in the transactions of the society, recently issued.

Since the ordinary channels of communication with the public are closed, the educational work of the society has been attempted through pamphlets, tracts, leaflets and through conferences and lectures in schools, colleges, settlements and in various social organisations. The instructors in the physical training department of the Y. M. C. A. have shown a most cordial willingness to co-operate in this educative work. Many teachers, instructors and pedagogists in various institutions throughout the country have exhibited, by letters of inquiry and commendation, a deep interest in this movement. Charity organisations, humane societies and women's clubs are becoming interested. While physicians generally have not responded to the demands of this work with that spontaneity and enthusiasm which was hoped for, yet the best element in the medical profession and a large and influential lay element have become members of the societies organised in various cities.

The progress of this movement cannot fairly be measured by the results of actual accomplishment. Seed has been sown which, it is believed, will germinate and bear fruit later on. "Education Within the Medical Profession," which formed the subject of one of the first papers read before the society, is actively going on through papers and discussions in medical societies, clubs and associations, and many physicians who had not kept pace with the recent advances made in our knowledge of the social dangers of these diseases are now becoming impressd with the importance of this work. One result of this movement which serves to show the breach made in the walls of traditional prejudice is illustrated by this gathering here. Thanks to the change in professional and social sentiment, we have now the courage to bring out in the open this forbidden topic which the medical profession has always discussed behind closed doors, to show this social scourge to the public face to face, to even pronounce its name without fear of shocking the sensibilities of a public audience.

Finally, the work in which your co-operation is solicited is difficult, and, there is no disguising the fact, is unsavory, even distasteful. It will never receive the endorsement of fashion or society, nor gain the plaudits of the multitude; popularity will never perch upon its banners; it can be undertaken only from a sense of duty. This duty imposes upon the medical profession a responsibility which cannot be evaded, and upon all those interested in the social welfare a moral obligation which cannot be disregarded. It is a work not only in the interests of preventive medicine, but for the good of humanity, and, in the words of the illustrious Pasteur, "when there is question of good to be done, our duty ceases only with our ability to do more or do better."

